# Evaluation of the Effectiveness of Remote Monitoring to Establish a Community Health Intervention During COVID-19: A Community Intervention Trial

**DOI:** 10.1089/tmj.2022.0118

**Published:** 2023-02-06

**Authors:** Wanyu Ji, Wenjing Shi, Xiaodong Li, Xia Shan, Junchao Zhou, Fengyuan Liu, Feng Qi

**Affiliations:** ^1^ Xinglin College, Nantong University, Nantong, China.; ^2^ School of Public Health, and Nantong University, Nantong, China.; ^3^ Medical School, Nantong University, Nantong, China.; ^4^ Department of Pharmacy, The Fourth Affiliated Hospital of Nantong University, Yancheng, China.

**Keywords:** electronic health records, telemedicine, COVID-19, teleconsultations, remote monitoring, telehealth

## Abstract

**Background:**

The widespread use of telemedicine systems and medical information networks has made telemedicine one of the current trends in health care. The purpose of this article is to propose a community health intervention with remote monitoring and teleconsultation during COVID-19 for the prevention and control of COVID-19 at the rural level.

**Methods:**

In this intervention study, a randomized group of 1,500 community residents was selected. A questionnaire with acceptable validity and reliability was used to collect data. The study was conducted with the test group itself as a control, and the questionnaire was completed again 6 months after the health intervention through remote monitoring. The extent of the effect of the remote monitoring intervention on community health during COVID-19 was measured. The data were entered into SPSS 26, and the data were analyzed using the K–S normality test, t-test, and chi-square test.

**Results:**

After 6 months of the intervention, the differences in mean scores of the test group were statistically significant (p < 0.05) in cognition, perceived benefits, self-efficacy, and behavioral outcomes, with a substantial increase in mean scores for all variables.

**Conclusions:**

The application of remote monitoring during COVID-19 in rural communities can influence the health perception, benefit perception, self-efficacy, and behavior of community residents, thus effectively preventing and controlling the spread of COVID-19 in rural communities. It reduces medical barriers for rural areas while meeting.

## Background

The widespread use of telemedicine and medical information networks has led to telemedicine becoming one of the current trends in health care.^1,2^ Telemedicine uses communication and information technology to provide medical services to patients from a distance.^3,4^ It contains many subtypes, of which remote monitoring and teleconsultation are essential parts of telemedicine. The former refers to wearable or mobile devices to monitor patients or allow patients to report to their providers through the internet or telephone to track their health status or disease progress closely.

The latter refers to creating an entirely new connection between medical specialists and patients so that patients can receive consultations with and treatment and care under the guidance of remote specialists in their original location, in their original hospital. Such cross-regional real-time consultations can save doctors and patients a lot of time and money, as in the case of remote intensive care unit care, emergency room consultation applications, or collaboration between information providers and specialists outside of geographic boundaries.

They enable off-site “face-to-face” consultations between specialists and patients and specialists and medical staff through the remote transmission of data, text, voice, and image information. The combined application of the two provides a solid foundation for medical treatment toward regional expansion with a convenient and reliable new medical treatment.^5^ They can be new guidelines and tools for improving the medical service and exchanging experiences in medical services.^6^

During the COVID-19 pandemic, nonmedical interventions such as lockdown and home isolation were implemented nationwide. This effectively reduced the chance of virus transmission and kept the population safe, rather than increasing the risk of cross-infection from COVID-19 with off-site visits.^7^ Long-term home isolation affects the physical and psychological health of the people, but the restricted range of movement of the population makes it difficult to access medical care.^8–10^ Therefore, primary care needs to make full use of telemedicine systems and strengthen health informatization. This will help break through the time and space limitations of people's medical consultation during COVID-19 and provide convenient medical and health consultation services for the people.^11^

Unequal distribution of health care is also an important cause, and it makes it difficult for people to access health care.^12,13^ According to the survey, in terms of the current development of medical and health care, the conditions of medical resources in rural areas lag behind those in urban areas. The accessibility and efficiency of health care services in rural areas are low.^14^

In addition, the prevention and control of COVID-19 in rural areas are not as vital as that in urban areas, mainly because the basic health information of rural residents is missing or not updated quickly. This is not conducive to the comprehensive prevention and surveillance of COVID-19 in rural areas.^15^ We encourage the complete application of remote monitoring and teleconsultation in rural areas. This can assist in preventing and controlling COVID-19 while addressing the difficulty of accessing medical care for the population during COVID-19.^16^

This article aims to propose remote monitoring and teleconsultation intervention for preventing and controlling COVID-19 in rural areas. It can collect health information of rural residents and exchange consultations between doctors and patients without compromising the security of medical information.

## Methods

### STUDY DESIGN AND SAMPLING

This study was conducted in Nantong City, Jiangsu Province, China, using a randomized whole-group sampling method. Four communities were randomly selected from the area. Residents were then randomly selected as participants to the study group from these communities. The number of residents who participated in this study was 1,500.

### INCLUSION AND EXCLUSION CRITERIA

Inclusion criteria for the study included permanent residence in the community, absence of psychiatric history and cognitive impairment, voluntary participation, and informed consent of the person or guardian. Subjects' unwillingness to participate at any stage and leaving the community were considered exclusion criteria.

### MEASURES

A professionally designed questionnaire was used for the study data collection, and the validity and reliability of the questionnaire were determined. A Cronbach α internal consistency method with a sample of 300 was used. The reported coefficients of knowledge reliability were α = 0.82, benefit assessment α = 0.87, self-efficacy assessment α = 0.87, and behavioral outcome assessment α = 0.88 (*Table 1*).

**Table 1.** Overview of the Content of the Remote Monitoring Intervention Survey Evaluation

MAJOR PROJECTS	NUMBER OF ITEMS (FORMAT)	SCORING CRITERIA (RANGE)
**(1) Knowledge:** refers to the theoretical or practical understanding of remote monitoring	5 items (True - false - don't know)	“Correct” response = 2, “don't know” response = 1, “incorrect” response = 0 (0–10)
**(2) Perceived benefits:** refer to the individual's perception of the positive aspects of remote monitoring	14 items/5-point Likert scale (strongly disagree to strongly agree)	Strongly disagree = 1, disagree = 2, don't know = 3, agree = 4, strongly agree = 5 (14–70)
**(3) Self-efficacy:** the individual's perception of his or her ability to perform the behavior successfully	5 items/5-point Likert scale (strongly disagree to strongly agree)	Strongly disagree = 1, disagree = 2, don't know = 3, agree = 4, strongly agree = 5 (5–25)
**(4) Behavior:** refers to preventive behavior related to COVID-19	8-item/5-point Likert scale (always to never)	Always = 5, often = 4, sometimes = 3, rarely = 2, never = 1 (8–40)

The questionnaire consisted of two parts. Part I: Basic information about age, gender, etc. Part II: Remote monitoring of health intervention effectiveness assessment (including knowledge, benefit assessment, self-efficacy, and behavioral outcomes).

Participants assessed the benefits of remote monitoring regarding medical services, information systems, financial costs, and psychological care. Individuals' perceptions of their ability to take health measures during the COVID-19 constituted a self-efficacy assessment. These questions were answered in the survey using a 5-point Likert scale. The study was conducted with the test group itself as a control, and the questionnaire was completed again 6 months after the educational intervention. The degree of effectiveness of the community health of the remote monitoring intervention during COVID-19 was measured.^17^ A community-based base station was established, and professional staff conducted community data collection and intervention supervision.

### HEALTH INTERVENTIONS

**Fig. 1.**
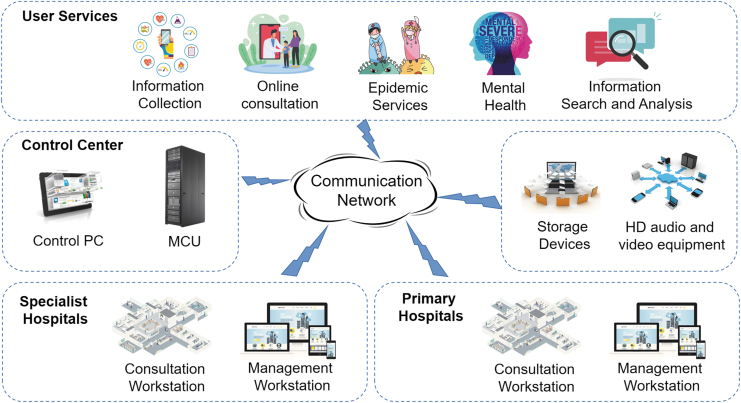
Remote monitoring system overview.

Community health service centers are equipped with intelligent examination and monitoring equipment and established an internet management platform—dynamic monitoring and treatment guidance. Community residents can use cell phones, computers, and other electronic devices to instantly transmit health data to the community health monitoring and management platform, forming dynamic electronic health records (*Fig. 1*). Significantly during COVID-19, the communication and positioning between the cell phone and the base station can help obtain the residents' location information and travel history.

**Fig. 2.**
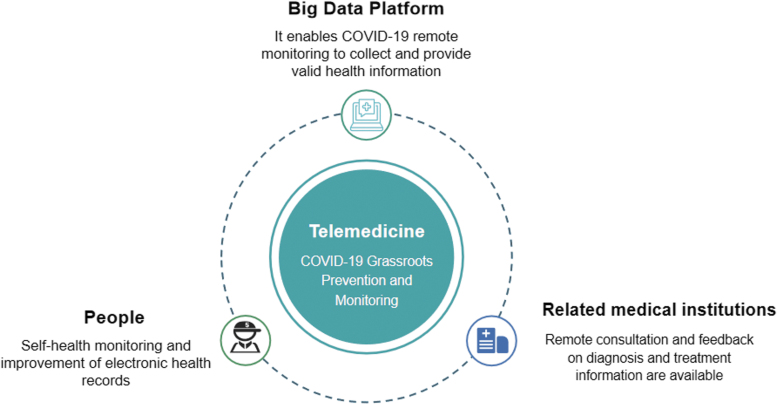
Remote monitoring main structure.

This part of information can be viewed at any time of the day through mobile electronic devices such as cell phones. In contrast, body temperature and person contact are important observations that are potentially risky and difficult to monitor for COVID-19. In this intervention, participants were asked to spend a little time filling in the health information on their cell phones once a day. For the elderly living alone, left-behind children, and other people with difficulties using intelligent devices, community control staff of local community service stations will provide health information filling household monitoring services for this group of people after receiving standardized training (*Fig. 2*).

**Fig. 3.**
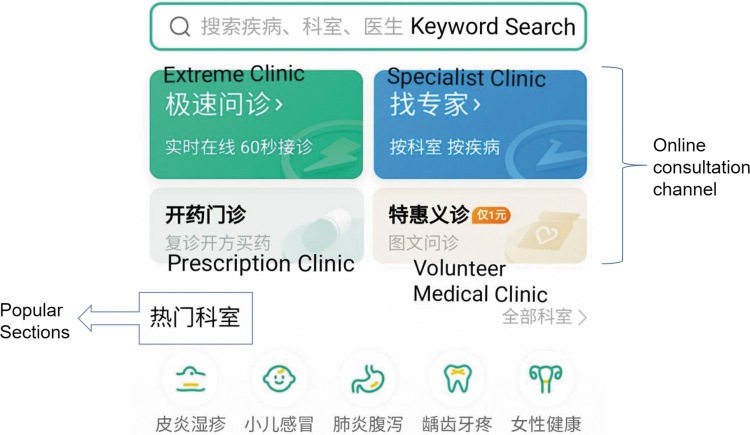
Teleconsultation system user interface.

**Fig. 4.**
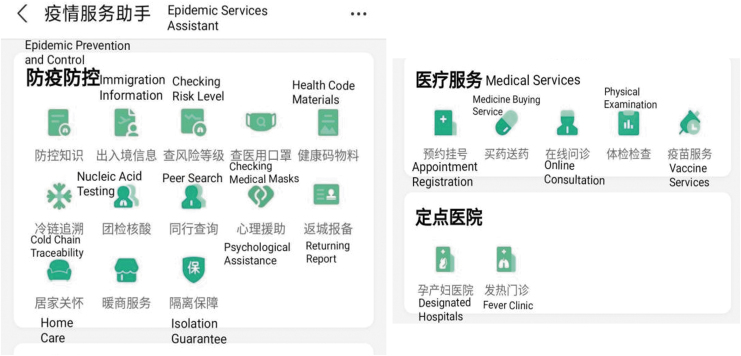
Access to special medical services during COVID-19.

The family doctor is responsible for data inquiry, feedback, observation, and guidance of body temperature and other critical body mass index values determining the condition, prescribing treatment and health prescription, adjusting the treatment plan, and regular remote consultation (*Fig. 3*). In addition to conventional treatment measures, remote health management interventions are implemented based on dynamic health monitoring information. For suspected infected patients during COVID-19, relevant medical institutions provide online emergency fever clinic services (*Fig. 4*). The hospital will contact nearby isolation sites for isolation and observation if necessary.

Through the remote health monitoring platform, the family doctor or specialist collects and analyzes the empirical data of the participants, formulates personalized treatment plans and lifestyle interventions according to the physical condition, tracks each participant in the experiment in real-time, monitors dynamically, and adjusts the medication according to the changes in the state, and provides comprehensive management interventions through remote consultation, SMS reminders, telephone guidance or face-to-face health education, and distribution of promotional materials. The interventions were managed comprehensively through remote consultation, SMS reminders, telephone guidance, face-to-face health education, and promotional materials.

Community hospitals set upgraded treatment offices to implement graded management services. Measures include weekly face-to-face health checkups or health promotion by experts on various risk factors. For example, to stop smoking and limit alcohol, follow the principle of the energy balance of diet and exercise, make recipes, and guide the exercise methods and reasonable medication adjustment by the physical condition.

### STATISTICAL ANALYSIS

The study used SPSS 26.0 statistical software to analyze the assessment results. The Kolmogorov–Smirnov test examined the normality of the data. Since the data did not conform to a normal distribution, the Wilcoxon Signed Ranks Test was used to compare participants' scores on cognitive, benefit assessment, self-efficacy, and behavioral outcomes before and 6 months after the intervention. The level of statistical significance was set at <0.05.

## Results

The study sample size was 1,500. There was no sample loss during the intervention, and participants completed the questionnaire completely and accurately (*Table 2*).

**Table 2.** Basic Information of the Participants (*N* = 1,500)

	n (%)	MEAN ± SD	p-VALUE^*^
Gender		1.42 ± 0.49	0.000
Male	877 (58.5)		
Female	623 (41.5)		
Age (years)		4.82 ± 1.41	0.000
18–24	144 (9.6)		
25–30	102 (6.8)		
31–40	299 (19.9)		
41–50	478 (31.9)		
51–60	288 (19.2)		
≥61	189 (12.6)		
Highest education		2.72 ± 1.32	0.000
Elementary school and below	316 (21.1)		
Junior high School	397 (26.5)		
High school/technical school	420 (28.0)		
University specialists	128 (8.5)		
Undergraduate and above	239 (15.9)		

^*^ Chi-square test.

SD, standard deviation.

The results showed that the remote monitoring intervention significantly improved people's healthy cognitive and behavioral efficacy in the grassroots community (*Table 3*).

**Table 3.** Pre- and Postintervention Comparison for Each Group

STRUCTURES	PREINTERVENTION MEAN ± STANDARD DEVIATION	POSTINTERVENTION MEAN ± STANDARD DEVIATION	MEAN DIFFERENCE	p-VALUE^*^
Cognitive awareness	6.35 ± 2.46	9.89 ± 0.51	3.54 ± 1.95	0.000
Benefits assessment	39.60 ± 7.33	59.90 ± 3.92	20.30 ± 3.41	0.000
Self-efficacy	16.08 ± 4.13	21.98 ± 1.89	5.90 ± 2.24	0.000
Behavioral outcomes	28.19 ± 5.30	35.93 ± 2.76	7.74 ± 2.54	0.000

^*^ Wilcoxon signed ranks test.

## Discussion

The main objective of this study was to propose the application of remote monitoring and consultation in primary communities and determine the impact of this community health intervention on residents' COVID-19 preventive measures during COVID-19. The study results showed a significant difference in the mean cognitive scores of the pilot group before and after the telemonitoring health intervention. The significant differences in cognitive scores stemmed from the impact of the community intervention guidance on residents' increased awareness, for example, the establishment of electronic health records, telemedicine services, and the availability of health education during the intervention.

Significant differences were found in the mean scores of subjects' benefit assessments. The remote monitoring intervention helps community residents achieve real-time monitoring and feedback of their health status, with quick access to information related to medical services and accurate and comprehensive content. Multiple researchers have confirmed the above.^18–21^

For example, residents can get feedback through cell phone terminals and check the valid COVID-19 monitoring status in real time. Under the same measurement criteria, healthy people will be represented by a “green” QR code. The QR code can be scanned to obtain detailed health information about the user. People with abnormal indicators or those living in low-, medium-, or high-risk areas will be represented by “yellow,” “orange,” and “red,” respectively. Participants said that this would help increase the identification of people at risk during COVID-19.

The teleconsultation service provided by the community hospital based on surveillance has vastly improved the efficiency of the consultation in the community hospital.^22–24^ This is because the registration at the hospital service window is missing. While effectively ruling out suspected cases, direct contact of personnel and the risk of exposure are avoided. Effective avoidance of cross-infection is an essential tool for primary outbreak prevention and control. Hospital visits during COVID-19 increase the risk of nosocomial cross-infection, which is highly likely to lead to the wide spread of COVID-19. The teleconsultation service of graded consultation can meet the needs of the home-isolated population for daily consultation.^25–27^

In addition, psychological support such as psychological assistance and in-home care during COVID-19 is also an essential element that needs to be considered.^28^ According to existing studies, the COVID-19 outbreak has brought different degrees of psychological impact to the population, affecting people's psychological perception, mood, and behavioral changes, such as panic, anxiety, and impatience.^29,30^ For healthy people, this stress may come from the disease prevention and surveillance situation, concerns about their own risk of infection, etc.^31^

The psychological impact may stem more from the disease progression and prognosis for patients. Real-time updated remote surveillance allows the population to follow better and keep track of the development of the epidemic and alleviate psychological stress.^32^ Remote monitoring and consultation ensure the accessibility of primary health care. It promotes the psychological health of the population during COVID-19 by taking advantage of online information technology and low cost to conduct online psychological counseling without threatening the health and safety of the people.^33,34^

Self-efficacy is a precursor to behavior.^35,36^ Therefore, special attention should be paid to improving self-efficacy. This study showed a significant difference in the mean scores of participants' self-efficacy before and after the intervention. The telemonitoring intervention in the community contributed to increased self-efficacy for COVID-19 prevention measures.^37^ There was a significant difference in the mean scores of the behavioral outcomes of the participants in this study after the remote monitoring intervention.

This suggests that remote monitoring and related services helped increase the community's behavioral intentions toward COVID-19 prevention. After the intervention, people were more willing to take the initiative to fill in health information, strengthen personal protection, and reduce going out. Enhancing health awareness and promoting health behaviors facilitate public health management during COVID-19.^38^

Through remote monitoring and control, remote consultation, and graded treatment, the quality of public health services has been significantly improved. The risk rate of COVID-19 exposure has been reduced to ensure the health quality of the community. The effect of remote real-time monitoring and management in COVID-19 early screening prevention is noticeable, and the compliance of residents' health monitoring behavior is significantly improved. At the same time, through the hospital management platform and cell phone terminal management, family physicians pay more attention to the follow-up and guidance of residents. They can promptly detect and deal with suspected COVID-19 infection cases.

Family physicians involved in this community intervention study reported that community-based remote monitoring and consultation during COVID-19 effectively assisted in community-wide prescreening and control of COVID-19. In addition, it helped family physicians to understand the health status of community residents in a timely and accurate manner, which vastly improved the efficiency and quality of their work. The remote monitoring combined the basic medical and public health service programs and effectively reduced the pressure on hospital visits and prevention and control during COVID-19.

The self-subjective reports used in this study were not subjected to rigorous quantitative investigation about assessing application effects. Therefore, the actual results are subject to bias, and these will be further improved in future studies. It should also be noted that the population interviewed in this study was mainly in the rural areas of Nantong, Jiangsu. For the areas with the most severe outbreaks of COVID-19, there may be other issues to be discussed. Our data need to be investigated and confirmed in future broader population studies, and we look forward to relevant studies by other research groups.

## Conclusions

In general, during the COVID-19 pandemic, telemedicine essentially reduced medical barriers in rural areas. Especially in COVID-19 monitoring, it has significant advantages due to safe and efficient information acquisition methods and transmission channels. At the level of remote diagnosis and treatment application, it improves the utilization of primary medical and upper medical resources using triage consultation. Therefore, this article has a good reference value for applying COVID-19 urban and rural telemedicine and provides a theoretical basis for active intervention in public health emergencies in the future.

## Ethics and Consent to Participate

The ethics committee approved the study of the First People's Hospital of Yancheng City [2020]-(K-061). Informed consent was obtained from all participants. The participants were duly informed about the purpose of the study. Moreover, the researchers assured them that the information collected would remain confidential and be used only for the analysis. By answering the questions in the questionnaire, the respondents indicated their agreement to participate.

## Data Availability

The first author can provide access to study data upon reasonable request after the study team has completed planned data analyses.
